# Report on Materia Medica and Therapeutics

**Published:** 1858-11

**Authors:** John Bell


### REPORT ON MATERIA MEDICA AND THERAPEUTICS FOR THE YEAR 1857.

By JOHN BELL, M.D.

---

### PART II.

### ALTERATIVES.

THE term alterative, for a particular class of medicines, furnishes a strong proof of the difficulty of classifying the articles of the Materia Medica, with a view of designating their therapeutical uses. Every remedial agent, whether medicinal or hygienic, is an alterative, since it causes some change or alteration in the organism. Commonly, however, we restrict this title to a substance which produces no immediate or physiological effect, such as purging, sweating, augmented sensibility, or mobility, but which brings about a gradual and salutary change, the only measure of which is in the ultimate restoration of the altered organ and deranged function to their normal states. But we cannot be said to have a class of medicines whose action on the animal economy is solely alterative; for, if taken in larger doses, most of them show other effects, and their affiliation with articles under another class. There is this advantage, however, in admitting the title of alterative with the signification usually attached to it, viz.: that the use of such an article saves the organism from the perturbing and not seldom damaging influence of the more heroic modes of treating diseases; at the same time that opportunity is allowed for nature, through the substantive form of the common agents of hygiene, to display her restorative powers. For our present purpose, we shall find it convenient to make use of the term "alteratives," not as an appropriate one to head a natural class, but as a string on which to hang our diverse items of therapeutical results—like so many parti-colored beads—which would otherwise be lost or hard to find at the moment when we wished to make use of them.

*Podophyllum and Leptandria.*—Dr. R. E. Haughton, of Indiana, in a paper, under the head of "New Remedies," (*Nashville Journ. of Med. and Surg.*, Feb. 1857,) tells us, that, with a view to save his patients from the harsh operation of calomel, including, at times, ptyalism, he resolved on trying substitutes milder in their operation, but equally efficacious in procuring the desired relief. He continues:—"There are cases which will not bear a blue pill without its specific action and in those cases I found that podophyllum, combined with leptandria, in the proportion of half a grain of the former to three or four grains of the latter, is a most reliable and efficient alterative, producing no nausea, pain, or unpleasant symptoms, and may be given in proper quantities, regulated (by the age of the patient) to all ages, from the infant to adult age, having no fear of any specific action set up in the glandular system, and of course evading the prostration consequent upon such conditions." The minute dose of the first of the articles here prescribed, coupled with the previous remarks of Dr. H. on the active principle of the *Podophyllum Peltatum*, (May apple,) having been obtained by Mr. Hodgson, of this city, and by him called podophyllin, leads us to believe that the author means this latter, instead of podophyllum, in the above prescription. The leptandria, which forms the other component part, is the active principle of the *Leptandria Virginica*, sometimes called Culver's physic. "It is an excellent alterative alone, producing augmented biliary secretion, and thus becomes a valuable adjunct to other remedies in the treatment of disease."

*Iodide of Potassium for the Cure of Syphilis.*—Dr. C. T. Bonsall, of Trenton, New Jersey, (*Med. and Surg. Reporter*, April, 1857,) relates the history of a case of syphilis in a married female, in which the use of iodide of potassium was eminently successful. The disease was of four years' duration. There was ulceration of the neck of the uterus, and continued discharge from the os tincæ; discharge from the urethra; inflammation and ulceration of the mouth and throat; nodal formations and rheumatic pains. Injections of ℥ij. of protosulphate of iron dissolved in a pint of water, were thrown up the vagina repeatedly, and five grains of iodide of potassium were given three times a day, with twenty [minims?] of wine of colchicum. On the 6th day the quantity of the iodide was doubled. On the 12th day, congestion and dryness, accompanied by pain of the conjunctiva, bronchial and nasal mucous membranes, commenced, all of which passed away with catarrhal discharge. On the 21st day, fifteen grains of iodide of potassium were added to the former allowance, and from this time her disease disappeared rapidly. During the first week in May her menses returned; she was becoming quite fleshy, and seemed wholly cured of her disease. The treatment by Dr. Bonsall had been begun on the 22d of February.

This poor woman might be supposed to have suffered enough, but it was ordered otherwise. The graceless husband communicated to her fresh disease in the last week of August. Dr. Bonsall, on being called in, found indurated chancres in the vagina, internal labiæ, and in the orifice of the urethra. Several buboes had begun to develope themselves on the groin. The chancres were immediately destroyed by caustic, and the eroded surfaces healed promptly; but there now commenced a rapid and fearful development of secondary syphilis. We omit the details, to pass to the treatment—administration of the iodide of potassium—which was again successful. At the beginning, thirty grains of the iodide were given daily in three portions, taken in half a pint of hop tea. Veratria was substituted for the wine of colchicum, which was found to act too freely on the bowels. "On the 15th of October she was taking sixty grains of iodide daily, and half a grain of veratria; but the disease was still progressing. On the 25th the dose reached a hundred grains, and three-fourths of a grain of veratria. On the 1st of November she was taking one hundred and twenty grains of the iodide, and a grain of veratria. About the 6th, iodism began; a profuse secretion of tears, sneezing, and an undue quantity of saliva thrown out by the salivary glands, told that the limit had been reached, beyond which it was not safe to give iodine." The disease had now yielded in every part. After the 25th, the dose of iodide was gradually diminished, and left off entirely in the latter part of December. The general health of the patient rapidly improved, and she gained flesh.

*Iodide of Potassium Injection in Leucorrhœa.*—In the *New Orleans Med. and Surg. Journ.*, a solution of the iodide of potassium, ʒiss., to ℥viii. of pure water, has done good service, in the form of injections, which are to be introduced into the vagina three or four times a day. We would recommend as an adjuvant to this treatment, and also as often a preventive, and sometimes itself curative, regular twice daily injections of castile soap and water into the vagina.

*Chlorate of Potash Injections in Leucorrhœa and Ulceration of the Os Uteri.*—Dr. Bedford Brown, of North Carolina, gives an account of two cases (*Am. Journ. Med. Sci.*, July, 1857) in which he used injections of the chlorate of potash in solution, ʒj., to ℥viii. of water, as an injection in the above diseases, with the best results. The injection to be used twice daily. Dr. Brown mentions a case of gonorrhœa in a female cured by the same remedy.

*Chlorate of Potash in Mercurial Stomatitis.*—Additional evidence in favor of this treatment is furnished by Dr. Gallaher, one of the physicians of the Western Pennsylvania Hospital, Pittsburg, (*American Journ. Med. Sci.*, July, 1857.) "Judging from past experience, (says Dr. G.,) I now, with the use of this salt, can remove a mercurial disease of the mouth in from six to ten days, which, under any other proposed plan of treatment, would last from four to six weeks." His method of treating the patient is as follows: "He is placed in a warm and comfortable apartment, and made to live on gruel. I then order him ten grains of the chlorate of potash, dissolved in a tablespoonful of cold water, three or four times a day, according to the severity of the affection. Should there be ulceration of any portion of the mucous membrane of the mouth, I direct a weak solution of the salt to be applied to the denuded part several times a day; generally, nothing else is required, the cure being accomplished in a few days."

In *Stomatitis Materna*, Dr. Faulkner (*Virginia Med. Journ.*, December, 1857) speaks very highly of the chlorate of potash.

*Nitrate of Silver in Catalepsy.*—A singular case of this disease was treated successfully by Dr. Wm. R. King, of Louisburg, N. C., (*Am. Journ. Med. Sci.*, January, 1858,) in the month of May, 1857, by the use of the nitrate of silver. The tonic spasm and fixed rigidity of the limbs would be brought on by pressure on the nape of the neck, where there was considerable tenderness, also on the crown of the head, over a space about the size of the palm of the hand. Sweeping the floor, patting the feet, sawing wood, and all harsh or grating sounds, from whatsoever cause proceeding, had the same effect. The treatment adopted by Dr. King, was chiefly to administer one-quarter of a grain of the nitrate of silver three times a day, and gradually to increase it to a half grain; and also use counter-irritation with strong croton oil liniment over the tender vertebræ. Under these means the patient entirely recovered from all nervous disorders. Headache, or cerebral disturbance, was met by small doses of blue mass, followed by a saline cathartic or castor oil. The strength and general health were to be improved by some mild chalybeates.

*Iodine in Ovarian Tumors.*—Dr. Roemaer, of Otter Bridge, Va., relates (*Am. Journ. Med. Sci.*, April, 1857) his successful treatment of ovarian tumor by the use of iodine, both internally and externally. His formula for the former was—℞. Iodin., ʒi.; Iodid. potass., ʒij.; Aquae. destillat., f℥vi.—♏. Dose, a dessert-spoonful four times a day. A pretty strong dose of iodine to be thus repeated—rather more than three grains, or twelve grains in a day. The external application was made by painting the surface of the abdomen, "between the pubes and the umbilicus, to the left, toward the groin, twice daily." In the second case the compound tincture of iodine was given in tablespoonful doses, four times daily—equal to twenty grains of iodine in the course of the day. In the four cases thus treated, the ovarian enlargement was entirely removed by the means just designated.

*Nitric Acid in Pertussis.*—Dr. Charles Witsell, of Cheeha, South Carolina, gives an account (*Charleston Med. Journ. and Rev.*, January, 1857) of his success in treating pertussis by the use of nitric acid, as recommended by Dr. McNally, of Tennessee. He administered it sweetened, and diluted so as to resemble lemonade: of this, the nurse was directed to give the patients as much as they could drink.

*Creasote in Dysentery and in Cholera Infantum.*—Dr. Wm. H. McMate, of Arkansas, lauds (*Southern Med. and Surg. Journ.*, October, 1857) the curative powers of creasote in dysentery; but with what justice, may be inferred when it is known that the formula which he is in the habit of using, and on which he relies so much, contains a good share of morphia in addition to the creasote. It is as follows:—℞. Creasote, gtt x.; Acetic acid, gtt xx.; Morphine sulp., gr. ii.; Water, pure or distilled, ℥i.—♏. Of the above solution, the author is in the habit of giving "to an adult, a teaspoonful every two or three hours, until it checks the blood and mucus in the evacuations from the bowels." It will be seen that the patient takes, at each dose, a quarter of a grain of the sulphate of morphia, which, of itself, must count largely in the therapeutic agencies enlisted on the occasion. We find, moreover, on reading the histories of the cases treated by Dr. McMate, that calomel and Dover's powder, "in broken doses, were prescribed in alternation with the creasote."

Dr. W. K. Bowling, in the *Nashville Journ. of Med. and Surg.*, Aug. 1857, after alluding to Dr. Elliotson's recommendation, made many years ago, of creasote as a remedy for nausea and vomiting dependent on irritation, tells us that he holds this medicine to be superior to the means usually employed in acute cholera. His customary prescription is—℞. Mucilage of gum arabic, ℥ij.; Creasote, gtt iii.—♏. A teaspoonful every six hours.

*Nitrate of Potash and Cold Water Enemata in Dysentery.*—Dr. Henry Tiedemann, of Philadelphia, in a small tract "on Dysentery and its Treatment, 1857," dedicated to the College of Physicians, gives his views of the pathology of the disease in some detail, and then proceeds to an enumeration of the remedies which are had recourse to by different physicians. He ends with a declaration of his confidence in the superior therapeutic value of nitrate of potash, or, as he terms it, "*nitrate of potassium*, (kal. nitr.)" Dr. Tiedemann tells us that he has given this salt in large doses, but he fails to make any specification of quantity, and mode, and intervals of its administration. The tract contains no clinical details, no history of a single case. *Locally*, he orders "immediately after each evacuation, no matter how often they occured, injections of pure cold water." These he continues until the tenesmus ceases, which in the majority of cases happens in from six to twelve hours: "as the tenesmus diminished, the mucus and bloody evacuations also diminished, and when it ceased, they generally disappeared entirely." For diet he orders "milk, gruel, barley, rice-water, toast and water, and pure water—buttermilk, as much as the patient liked to take."

He looks to the state of the digestive organs, with a view to ascertain whether an emetic or a purgative be required before nitre is administered. "If, during the treatment with nitre and the injections of cold water, evacuations of fecal matter did occur, at least once in twelve hours, which usually was the case, I recommended a corresponding dose of castor oil." Dr. Tiedemann asserts that this treatment is applicable, "in the two first stages of the disease, to every age, sex, constitution; every mode of living, in every epidemic, every season, every so-called species of the disease." After this strong, unqualified claim of entire success under every possible contingency, the reader must feel somewhat surprised at the author's admitting that he is "not acquainted with the tropic, cramp, and so-called other dysenterics, except from reports,"—a tolerably large drawback thus it seems to us, from the validity of a position assumed in so unqualified a manner.

When the dysentery proper has disappeared, which in his hands is often accomplished in twelve hours, he gives a solution of sulphate of quinine, and on the third day often allows a better diet to the patient; the injection is to be continued, after each evacuation, for a few days longer; and if regular evacuations do not take place, castor oil is to be taken from time to time. In very severe cases, particularly in hot weather, he orders "ice water to be injected after each dysenteric discharge, which relieved the patient almost immediately."

The author of the tract defines the third stage of dysentery, over which he does not assert control by his favorite treatment, to exhibit "extensive and deep ulceration of the mucous membrane, and undermining ulceration of the submucous tissue. *Discharge.*—Blood mixed with pus, shreds of necrotic cellular tissue, and ichor."

*Phytolacca Decandra—Poke Root—in Granular Conjunctivitis.*—The readers of the *North American Med. and Chir. Review*, Jan. 1857, need scarcely be reminded of the valuable paper of Dr. C. S. Fenner, of Memphis, Tennessee, on granular conjunctivitis, and of the high opinion which its author entertains of the phytolacca, in the form of tincture or of strong decoction, in this disease.

Dr. Parry, of Indianapolis, had, at a previous period, made use of the poke root in the case of a patient suffering under rheumatism conjoined with conjunctivitis. In a short time both diseases yielded to this treatment.

*Oxide of Zinc in Night Sweats.*—Dr. S. J. Abbot, of Boston, communicates, in *Boston Med. and Surg. Journ.*, April, 1857, the results of his numerous observations on the excellent effects of the oxide of zinc in night sweats. He adopts the prescription given by Dr. Theophilus Thompson in the *London Lancet* for October, 1854, viz.: four grains of the salt to three grains of extract of conium or of hyosciamus, to be given in two pills, which are to be taken at bed-time. Dr. Abbot has so much confidence in the remedy, that he always prescribes it first in preference to any other, and he rarely meets with disappointment. He introduces a synopsis of no less than twenty-three cases, in which he gives a condensed statement of the auscultatory signs in each, the duration of the disease, &c., so as to show the stage of the latter at which the remedy was administered. The remedy had been well spoken of a year or two before, by Dr. J. B. S. Jackson, and by Dr. Wm. E. Coale. The cases recorded by Dr. Abbot are all, with two or three exceptions of phthisis. Dr. Jackson, in afterwards adducing his own experience in confirmation of Dr. Abbot's, adds, that he has found the remedy equally serviceable in sweating in other diseases besides phthisis; as in intermittent fever, for instance. We once suffered from this occasional sequela of this fever: it was either removed, or wore itself out by continuing to use the bark, with the addition of elixir of vitriol.

Some substitute for the narcotic extracts already mentioned the extract of belladonna, in a dose of two grains, combined with four of oxide of zinc.

Dr. E. J. Coxe, of New Orleans, thinks most favorably of the sulphate of zinc according to the formula prescribed by Dr. Barlow, (Practice of Medicine,) viz.: one grain united to four of the extract of hyosciamus, to be made into a pill, and taken every night at bed-time.

*Iodide of Potassium in Asthma.*—A writer in the *Boston Med. and Surg. Journ.*, October, 1857, over the initials of C. S. S., tells of his having employed the iodide of potassium in a number of cases of asthma, without having been disappointed in its efforts in a single case. As far back as 1847, Dr. Casey, as we learn from this writer, in an article in the *Boston Journal*, speaks in laudatory terms of "the unequivocal and decided relief obtained from exhibiting this medicine." From the same source we learn that there is a patent medicine manufactured in Boston, and sold wholesale and retail by druggists of that city, which has obtained a reputation as a specific in asthma. An analysis of a bottle of this, by C. S. S., showed that iodide of potassium was one of its chief ingredients.

"*Head Matter*" *and Cacao Butter as Substitutes for Cod-Liver Oil.*—Dr. G. P. Camman, of New York, in a note addressed to the editors of the *New York Journ. of Med.*, January, 1858, directs attention to the oily substance taken from the cavities of the head of the spermaceti whale, known in commerce as the "head matter." In summer it presents the appearance of oil, with a copious white fleecy sediment; but in winter, when chilled, it resembles imperfectly frozen ice cream or beef drippings. It may be obtained from the manufacturers of sperm candles, and should be used while fresh, (in its crude state as landed from ship board,) for it becomes rancid by being kept. It is preferable to cod-liver oil on account of its being more agreeable to the taste, leaving a pleasant flavor in the mouth, and also from its being more nutritive and soothing. It is less apt to disagree with the stomach, and does not cause offensive eructations. The patient may take it either pure, in coffee, or with bread, boiled rice, potatoes, &c. When required, paregoric and syrup of the iodide of iron may be added.

A correspondent of the *Boston Med. and Surg. Journ.*, October, 1857, recommends cacao butter,—a solid oil, yielded in very large amount by the cacao-nut,—as a substitute for cod-liver oil. The suggestion was made, originally, by Dr. C. J. B. Williams, of London.

*Electro-Magnetism in Amenorrhœa and in Chronic Rheumatism.*—Dr. E. P. Christian, one of the assistant editors of the *Peninsular Journ. of Med.*, in a report at the annual meeting (1857) of the Michigan State Medical Society, on "some of the therapeutical applications of electro-magnetism," gives a case of amenorrhœa of several months' duration, associated with inveterate ague, in which the recovery was complete by the daily use of this agent for about a month.

A case of "an anomalous form of chronic rheumatism" was cured by the same means, between the 2d of April and 1st of May. We shall soon have to speak of this remedy, directed by the same hand, in intermittent fever.

*Esquimaux Treatment of Frost Bites.*—Under the present heading of "Alteratives," we may as well notice the above-named treatment, which is conducted by poor savages on as philosophical principles as might be supposed to emanate from any College of Physicians. Dr. Hays, the companion of Dr. Kane, in the last Arctic voyage, was the narrator, before the Ohio State Medical Society, at its meeting in Sandusky, June, 1857.

Dr. Hays gave a case coming under his own notice. "An Esquimaux had his leg frozen above the knee, stiff, colorless, and, to all appearance, lifeless. He was placed in a snow house at a temperature of 20° F. below zero. The parts were then bathed with ice-cold water for about two hours, then enveloped in furs for three or four hours. These frictions were used first on the feathery side of a bird skin, then with snow, alternately wrapping the limb in furs, and then rubbing it for nearly twenty-four hours. It was then carefully wrapped up, and the temperature of the snow house elevated by lamps above zero. On the third day the patient was taken to his house, (where they often have a temperature of 70° or 80° F.,) and in seventy hours he was walking about, with only a slight frost bite on one of his toes."

*Fluid Extract of Pareira Brava in Enuresis.*—The *New Orleans Med. News and Hosp. Gaz.* for July, 1857, quotes from the *Southern Journ. of Med. and Phys. Sci.*, a notice of a case of "piss-a-bed" in a young man twenty-five years of age, in which the fluid extract of pareira brava, in doses of a teaspoonful, three times a day, acted, to use the words of the patient, "like a charm." He was entirely relieved after taking a few doses.

### PURGATIVES.

*Podophyllum Peltatum—May Apple.*—One would hardly look for a knowledge of the doings of the Western Clinical Infirmary of Philadelphia in a medical journal, published in Keokuk, Iowa, and yet such is the fact. What follows may be read in connection with the remarks of Dr. Haughton on the alterative properties of podophyllin, in a preceding article, under the head of "Alteratives."

The reporter in the present case is Dr. William H. Letherman, then Resident Physician at the Infirmary. He "tried and experimented [with the podophyllin] during the last six months upon 400 patients, and always with the same result, as to its utility and beneficial action on the economy, in which hitherto the hyd. chlor. mit. was employed." He relates the case of a man, aged 25 years, who had suffered much from intermittent fever, and who, when he came under treatment, felt great pain, on pressure, over the region of the liver; his spleen was also enormously enlarged. The treatment consisted in the application of scarifying cups on two different occasions, and leeches at another time over the region of the liver. With these was conjoined, on the first day, the administration of podophyllin, in a dose, morning and evening, of half a grain, mixed with a quarter of a grain of carbonate of potash. No person can accuse the prescriber of giving too large a quantity of the latter article, added by him to correct acidity of the stomach, which prevents, we are told, the podophyllin from acting on the liver. No grounds are given for this opinion. The bowels were opened freely during the night, but we are not told how many of the four powders prescribed, each of them being of the strength just mentioned, were taken. On the following day, Dr. L. prescribed two grains of podophyllin and one grain of opium, with ten grains of sugar, to be divided into four powders; of which one was to be taken three times a day. No report is made of the effect of the powders on the bowels, except that we are told, three days later, "the bowels not being operated much, and the head remaining heavy, dull ache across the frontal region," a new prescription was filled up, consisting of podophyllin ten grains, carbonate of potash two grains, white sugar ten grains: the compound to be divided into twenty powders, one to be taken three times a day. We learn that two days afterwards, the patient "was able to be up, and says that he feels like a new man;" the right side free from pain; has no headache; bowels open three times a day; the discharges very dark and fetid; the liver reduced almost to its normal size. The subsequent treatment consisted in the application of tincture of iodine over the region of the liver and spleen for eight days—and the use internally of nitro-muriatic acid, in doses of 10 drops three times a day.

The reader will be struck with the singularly minute dose, approaching to the homœopathic, of the carbonate of potash, prescribed by Dr. Letherman; and he may ask: whether this is a case to illustrate the curative powers of the podophyllin?

*Cathartics in Dysentery.*—Under this title, Dr. O. C. Gibbs, of Frewsburg, N. York, communicates his ideas on the subject, in the *Buffalo Medical Jour-*

*nal* for Jan. 1857. The gist of his paper is a recommendation of the super-sulphate of magnesia in dysentery. The formula which he uses is that given by Dr. Dorsey in the June number of the *Western Lancet*, 1855, and which was recommended to the latter by Dr. Lemoyn, of Washington, Pa. It runs as follows:—Take of saturated solution of sulphate of magnesia *seven fluidounces;* aromatic sulphuric acid one fluidounce. Mix. The saturated solution is made by dissolving Epsom salts in an equal quantity of water, at 60° F. It will be ready for use in eight or ten hours. It should be shaken occasionally. The medium dose for an adult is one tablespoonful, in two or three ounces of water, every four to six hours, until it gently moves the bowels. Dr. Gibbs directs this dose to be taken in the morning, and repeated every three hours until it operates. Both Dr. Dorsey and Dr. Gibbs advise that a small quantity of morphia—the former says a sixth of a grain—shall be given at the same time with the saline solution. This course is repeated daily, as long as it is indicated. During the remainder of the twenty-four hours, Dr. Gibbs gives ipecacuanha with morphia, or such other remedies as the circumstances of the case require.

---

### DIURETICS.

*Croton Oil in Dropsy.*—We step across the border to chronicle the success of Dr. J. L. Stevenson, of Stratford, Canada, in the cure of dropsy with croton oil, (*Med. Chron.*, or *Montreal Monthly Journ.*, Nov. 1857.) In the first case, one of acute anasarca, half a drop of the oil was directed every morning. After the second dose, the swelling began to abate, the urine increased in quantity, and the dyspnœa entirely disappeared. On the ninth, the patient, a female, aged 30 years, was quite well. The second case, that of a boy aged six years, was of anasarca after scarlatina, and then catching cold. The croton oil was given in one-third of a drop every morning. The patient was quite well at the expiration of two weeks' treatment. Dr. Stevenson believes that the effects of the croton oil are due more to its stimulating the absorbents than to its drastic properties, "for in the first case it only caused three or four evacuations every morning, and in the second never more than two; and I may state (he continues) that in neither of the cases did the oil cause any griping."

---

### FEBRIFUGES.

THIS title, which designates the therapeutical operation of a certain class of medicines, might seem to be preferable to those derived from changes wrought in the functions, and which, resting on a physiological basis, indicate an increase or diminution of the various secretions and excretions, and an excited or obtunded sensibility. But the title, while it designates an object to be attained, viz., the removal of a pathological state called fever, indicates no similarity, harmony, or natural affinities among the substances or agents, by the use of which we hope to reach this object. Thus, for instance, blood-letting is a febrifuge; so also is blistering, and so are the cold-bath, and the vapor and warm-bath, and opium and simple sedatives, and salines and tonics—between no two of which is there community of physical and chemical characters, and of immediate physiological operation. Still, we may speak of febrifuges, as a term, helping us to arrange in our memory a series of medicinal agents the knowledge of which has been furnished by experience, but which no *à priori* reasoning or scientific aid could have enabled us to throw into one group.

There are but few febrifuges for us to notice on the present occasion, and they are of doubtful value. Under the class of Sedatives, the veratrum viride was mentioned by some of its more sanguine advocates as a febrifuge of undoubted power. After what we have already said, it is not necessary to recur to this topic.

*Chlorate of Potash in Typhoid Fever.*—Dr. V. C. Taliaferro, of Atlanta, Georgia, in the *Medical and Surgical Journal* of that city, for March, 1858, strongly advocates the use of chlorate of potash in typhoid fever. As his paper does not come within the year (1857) of our review, we ought, perhaps, to restrict ourselves merely to noticing it in connection with, and in corroboration of, the opinion advanced on the same subject by Dr. E. C. Ellet, of Illinois, in the *North-West. Med. and Surg. Journ.* for March, 1857.

Dr. Ellet was led to a trial of this salt in typhoid fever by his experience of its efficacy in mercurial stomatitis, and, subsequently, in the case of his own child, who was greatly and alarmingly reduced by remittent fever, and to whom he gave the chlorate on account of sore mouth, fetid breath, &c. The recovery from the fever was complete. Dr. E. finds that the best and most reliable dose for an adult, in typhoid fever, is twenty grains every four hours, without interruption, so long as febrile symptoms continue or much sordes remain on the teeth and gums. Might not another condition have been added by the writer for persisting in its use, viz., until it obviously failed either to arrest the fever, or materially to abate it? The chlorate "possesses decided diuretic powers, always causing a free flow of limpid urine."

Dr. Taliaferro usually combines veratrum viride with the chlorate, as in the following formula:—℞. Chlorate of potash, saturated solution, f℥iv.; Tinct. of veratrum viride, ʒss.—♏. Of this the patient is to take a tablespoonful every three hours through the day, with an opiate at night.

Dr. Jas. Morrison read (Dec. 17th, 1857, before the "Pacific Medical and Surgical Association") a paper on the use of the chlorate in typhoid fever. He refers to Dr. Chew, as using it in this disease, in the Baltimore Infirmary, ten or twelve years ago. In the year 1847, Dr. Morrison, Resident Physician of that Institution at the time, saw seventy-two cases of typhoid fever, two only of which had a fatal termination.

*Diluted Acetic Acid in Scarlatina.*—Dr. B. F. Schenck, of Lebanon, Penna., gives his experience in the use of diluted acetic acid in scarlatina, as previously recommended by Dr. J. B. Brown, London, 1846. Dr. S. begins by presenting the contrasted results of the common method of treating the disease, and of the practice by administering the acid. The first exhibited a mortality of 1 in 8½ to 9—the other was 6 deaths in 60 cases. All this occurred in the same epidemic, nor was there any abatement of its violence during the period of the acid treatment. Neither Dr. Brown nor Dr. Schenck trust to the diluted acetic acid alone. Cauterization of the throat, rubefacients externally, an aperient of calomel, followed by castor oil, precede the use of the acid, in the simple variety of scarlatina. In the anginose or malignant, the same remedies are to be had recourse to, with the addition of freer cauterization, and resort to stimulants—brandy or port-wine every four to six hours, with arrow-root, beef-tea, or mutton-broth; morphia at bed-time; or, whenever restless, sponging with tepid water and vinegar. So far Dr. Brown. Dr. Schenck was still more free in the use of stimulants. He administered brandy in graduated doses, two or three times a day, *from the beginning* of the malignant form, or on the second or third day in anginose cases. In a case of cardiac disease, (pericarditis,) occurring in malignant scarlatina, blood-letting was resorted to with advantage. When this remedy is employed, it ought to be followed immediately by diffusible stimuli.

The acid mixture prescribed by Dr. Brown consisted of distilled vinegar, diluted, by the addition, to one part of this liquid, of seven parts of water; of which one ounce is to be united with half an ounce of syrup and four ounces of distilled water. Dose, two tablespoonfuls every four hours. "This mixture is to be continued throughout the entire duration of the case, and for one or two weeks afterwards, or until the desquamation is well over." The wine and brandy may be given even in the febrile stage without any harm resulting, when they are combined with the acid. Dr. Schenck directs "the strength of the acid to be increased according to the violence of the attack; in bad cases giving it as strong as the patient can bear it."

---

### ANTIPERIODICS.

THE chief one of our antiperiodics, quinine, or, more properly speaking, Peruvian Bark, is also our great febrifuge, and it might, together with others of the present class, rank under this head, were they not used under other circumstances of morbid periodicity without fever.

*Nitric Acid in Intermittent Fever.*—Dr. J. C. Thompson, of Roseville, Arkansas, (*Southern Journal of the Med. and Phys. Sciences*, August, 1857,) relates his successful trials of nitric acid in six cases of intermittent fever, in all of which the customary remedies had been used without curing the disease. In three of the cases, pills of sulphate of iron, aloes, and rhubarb were given in addition to the acid. In two others, blue mass was prescribed in conjunction with the acid; and in one case in which there was much menorrhagia, a powder, consisting of one grain of opium and two grains of sugar of lead, was given every two hours till the discharge subsided. The formula for the diluted acid is one ounce in six ounces of water; of this, the patient is to take one drachm in an ounce of water every two hours during the intermission, and also the pills as above.

Dr. Thompson recommends, as a prompt and permanent antiperiodic, the following:—℞. Nitric acid, f℥i.; Water, f℥vi.; Sulphate of quinine, grs. xxviii—M. The dose is not stated. It is probably the same as that of the diluted nitric acid mentioned above.

The Committee of the Ohio State Medical Society recommends a fair and impartial trial to be given of the nitric acid and sulphate of cinchonia, in intermittent fever. The Chairman has had considerable experience in the use of the acid, since his attention was first called to it by Dr. Mendenhall, of Cincinnati, in 1854. In 35 cases out of 36, the acid was, in his hands, successful.

*Sulphate of Cinchonia.*—This salt has of late engaged a good deal of the attention of physicians, as a substitute for sulphate of quinine in the treatment of periodical fevers. Dr. J. M. Brannock, of Tennessee, (*Nash. Journ. of Med. and Surg.*, May, 1857,) states that, during the past season, he administered it in upwards of thirty cases, with results far exceeding his anticipations. The quantity given in a day varied from 20 grains to 2 grains, thrice in this period: the common dose was, at first, 4 grains, afterwards 2, at stated intervals. Dr. Brannock never saw the use of the sulphate of cinchonia to be followed by "the disagreeable head symptoms which render quinine, especially in large doses, so unpleasant in many cases."

Dr. Black, in "Remarks on the Causes and Treatment of Intermittent Fevers," (*West. Lancet*, Aug. 1857,) speaks well of the sulphate of cinchonia "when given in full and sustained doses, four grains every three hours until twenty-four are taken." The chief symptom, from its use, observed by Dr. Black, was a sense of cerebral fulness, with a strong somnolent tendency; and this only when given in unusually large doses. Dr. Black has directed, with advantage, a combination of the sulphate of cinchonia and chinoidine, as follows:—Six grains of the former to eighteen of the latter, together with ten or twelve drops of impure oil of black pepper, divided into twelve pills—two to be taken every two hours, beginning twelve hours before the expected chill.

*Asclepias Syriaca? The Root of Man—T. Plant—Milk Weed.*—Dr. Richard S. Cauthorn, of Richmond, Va., has written a paper in the *Monthly Stethsocope*, in which he calls attention to this plant as a useful remedy in intermittent fever. He has given it in six cases with success, and without its producing in any of them those distressing symptoms which often attend the administration of quinine. The bark of the root is the part used, and in the form of pills, preferably to that of tincture. Of the former, Dr. Cauthorn has given to the extent of from xviii grains to ℈i, and without producing either emesis or catharsis—results which he was told sometimes followed its use in domestic practice. Commonly, each pill contained two or three grains of the bark, which was sometimes combined with capsicum, sometimes given alone. A cure has followed the use of one dozen of the pills; two having been taken at intervals of every two or three hours.

*Gentiana Quinqueflora? Indian Quinine—Ague Weed.*—Dr. E. P. Wood, of Wisconsin, states that this plant is used to a very great extent in domestic practice, and that he has himself given it in quite a number of cases of intermittent fever with benefit.—*Trans. Illinois State Med. Soc.* for the year 1857.

*Electro-Magnetism.*—Dr. E. P. Christian, in a Report, already mentioned, and which we read in the pages of the *Peninsular Journ. of Medicine* for May,

1857, gives an encouraging view of the curative powers of electro-magnetic agency in periodical fever. He introduces cases of intermittent fever cured by it. His experience of its effects in diseases is summed up in the following series of propositions—1. No positive advantage could be discovered from the choice of one mode or direction for conveying the current over another. In the application intended to break up a paroxysm, one pole was usually placed against the epigastrium, the other being held in one hand; in dumb ague in the extremities, or neuralgic or rheumatic pains of these parts, the current was passed through them, along the course of the nerves. 2. Usually the force of the current, beginning with a weak one, was increased by degrees, as far as it could be tolerated without actual suffering, and continued, not arbitrarily, but until soreness was complained of from its use. 3. Local inflammation existing, there was invariably intolerance of the current, and acute phlogosis furnished a clear indication to abstain from its use. One case of bronchitis was invariably aggravated by this means; and in convalescence from pneumonia and in erysipelas there was absolute intolerance. 4. The benefit from electro-magnetism was the greater, the earlier the date at which it was resorted to after the attack; and the less was it to be relied on in cases in which there were extensive organic lesions. 5. The benefit derived from this agent was the most decided during the cold stage, on which occasion it almost uniformly checked the rigors, produced warmth and perspiration, relieved the pains in the loins and limbs, ameliorated the headache, and disposed the patient for a refreshing sleep. Sometimes the return of the fever was prevented altogether; at other times its duration was shortened, and it was made, also, lighter. The pulse was usually increased in fulness, as well as frequency, to the extent of from 8 to 12 beats. 6. The variations in this latter respect were greatest in those enfeebled by disease. 7. In two cases of chronic amenorrhœa, consequent upon ague, the menstrual discharge was brought on at the next regular period succeeding its use. 8. It displayed decided efficacy in resolving indolent tumors, enlarged glands, &c., besides reducing enlargements of the spleen. 9. Its too long or too frequent application caused not only soreness of the muscles but, also, a severe form of spinal irritation, requiring rest and counter-irritation for its relief.

The author very justly remarks, in conclusion, that electro-magnetism is a remedy of itself in a large number of cases, and an adjuvant in others; but that it is also powerful for evil as well as for good.

*Indian Corn Meal.*—Dr. D. B. Phillips, of the U. S. Navy, relates, in the *Am. Journ. of Med. Sciences*, Oct. 1857, his having given with success, in a case of intermittent fever, Indian meal, unsifted and uncooked, in the dose of a tablespoonful mixed in a glass of cold water, repeated every two hours. The first dose was taken in the last stage. Dr. Phillips gave his patient a dose of calomel the same night. On the following day, the use of the meal was resumed, with the effect of postponing the paroxysm, which was much milder, to a later hour. There was no return of the disease, although the meal was continued two days longer.

Some of our readers may ask,—Which was the curative agent in this case, the calomel or the Indian Meal?

*Prepared Chalk and Vinegar.*—With such a long list of cures, simple and complex, popular and professional, active and inert, it is wonderful that there should be found a single case of intermittent fever which is not immediately cured. Dr. Hodsden, of Sevier, Tenn., reports great success in the treatment of this disease with a mixture of prepared chalk and vinegar. It had cured, without a relapse, every case in which he had used it. He gave it in doses of a *tablespoonful* each (mixing them together and allowing the effervescence to cease) an hour before the time of the expected chill. The remedy operates as a cathartic, and excites the kidneys and other secreting organs. Dr. Hodsden received the knowledge of it from a friend in the West, who stated that he had seen hundreds cured by it.

It remains to be learned, whether a perfect acetate of lime will have the same effect as the imperfect one with a large excess of chalk, now recommended. Facts must always take precedence of theory; but, so far, general experience shows that all the remedies which have maintained their reputation for the cure of periodical fevers act on the nervous system; derangement of which is a fixed and predominent element in this class of diseases.

At the meeting of the East Tennessee Medical Society, in the transactions of which (*Nashville Journ. of Med. and Surg.*, June, 1857,) Dr. Hodsden's remarks are reported, Dr. Currey stated that he had used cider with satisfactory results in obstinate chills. Dr. Pride spoke of the good effects of sage tea in a case coming under his own observation.

*Confection of Cinchona.*—Dr. D. S. Gleninger, of Philadelphia, recommends highly, in the *Am. Journ. Med. Sciences*, Jan., 1858, this preparation (made of the calisaya bark) as one of our most efficient anti-periodics. "It sits well upon the stomach, does not produce the unpleasant cinchonism often complained of from sulph. quinia, etc., and comprises all the *powers* of the cinchona, gratefully disguised, and in such a concentrated form that the dose is *too small* even to be ungrateful to the most delicate stomach."

The formula furnished by F. L. John, pharmaceutist of this city, is the following: "*Confectio Cinchonæ*—℞. Cort. cinchon. calis. ʒj.; confect. sennæ, āā ℥i.; ammon. muriat. ʒss.; syrp. cort. aurant. ℥ij.—M. ft. confect. Dose, the size of a shellbark three times a day."

### TONICS.

*Quinine in Asthenic Pneumonia.*—The quinine in this case was not employed as an antiperiodic, and, therefore, according to usage, and in reference to its other effects, we place it under the head of "Tonics." Our own opinion of the *modus operandi* of quinine was given some years ago, when we had occasion to discuss the question of its remedial virtues in congestive fever. We will not go over the subject again in this place, but would merely remark that quinine, taking into consideration both its therapeutical and toxical effects, bears a closer analogy to the narcotic-sedatives than to any other class of medicines, certainly more than to tonics, as they are commonly considered and described.

Dr. J. D. Boyd, of Abbeville, North Carolina, in a note written in January of the present year to the editor of the *Charleston Med. Journ. and Review*, and inserted in its March number, states the satisfactory result of the administration of quinine in a case of asthenic pneumonia of several days' standing, in which "the patient had been purged and blistered according to the most approved modes." The state of the patient being one of extreme danger, in which dissolution was threatened, Dr. Boyd "determined to try the effect of large doses of quinine. Accordingly, five-grain doses of quinine were administered every three hours, and in less than twenty-four hours a marked change for the better was evident. The same treatment was continued for three days, during which time a rapid improvement took place, and the patient was discharged on the fifth day from my first visit, being then able to sit up."

*Iron in Erysipelas.*—Dr. W. Parker, of Richmond, Va., adds his favorable testimony (*Virginia Med. Journ.*, Nov. 1857,) to that of so many others, to the remedial powers of muriated tincture of iron (*tinct. ferri. chloridi*) in erysipelas. Dr. Parker recommends this medicine only in two forms of the malady, viz., 1st, in simple erysipelas of the head and face, and 2d, in what is perhaps properly called vesicular erysipelas. The first case narrated by Dr. Parker was that of an old lady, seventy-one years of age, of a gross habit and large frame. To her, after a dose of salts, he gave twenty drops of the tincture every four hours, night and day. On the next day the dose was increased to thirty drops, and two days after this it was reduced to twenty drops. Patient convalescent on the fourth day after he first saw her, and on the fifth of her disease.

---

### HÆMOSTATICS.

*Chlorate of Potash.*—Dr. Dupree, of South Carolina, informed his brother, Dr. Dupree, of Nashville, that he would find chlorate of potash, in doses of ten grains, three times a day, very useful in passive hemorrhage. He commends it especially in hæmoptysis. "At the time Dr. Dupree mentioned this to us, we had a case of troublesome menorrhagia of long standing, sent to us from an adjoining county, which resisted the usual remedies. The affection subsided under the chlorate, and the patient soon regained her usual health without any other remedy."—*Nashville Journ. of Med. and Surg.*, Aug., 1857.

*Oil of Erigeron.*—Dr. Currey, of Tennessee, reports, in the "Transactions of the East Tennessee Medical Society," (*Nashville Journ. Med. and Surg.*, June, 1857,) the employment of this oil in four cases of uterine hemorrhage; in three of which it had produced happy effects, and in one case of hæmoptysis. He administered the oil in the following state of combination:—℞. Ol. erigeron, gtt. xlviii.; Pulv. G. Arab. ʒij.; Sacch. alb., ʒij.; Water, ℥ij.—ℳ. Dose, a tablespoonful every one hour or two hours, according to circumstances. In the first case reported, viz., of hemorrhage in the tenth week of pregnancy, the discharge ceased entirely after four doses had been taken; and the patient went on to her full time without any untoward occurrence.

*Ergot.*—Dr. Affleck, at a meeting of the "Belmont Medical Society," (Ohio,) related a case of labor preceded by severe hemorrhage, in which ergot had a beneficial effect. Dr. Wierick thought it a good hæmostatic in hemorrhage following delivery.

*Ice.*—Dr. E. A. Hildreth, of Wheeling, Va., reports, in *Cincinnati Med. Obs.*, his success in checking a dangerous uterine hemorrhage by introducing ice into the cavity of the uterus. In the first case related, Dr. Hildreth, after other means, including cold affusion to the abdomen and ice on the hypogastrium and in the vagina, had been used in vain, introduced a lump of ice, as large as a lemon, into the uterus. "The effect was immediate and powerful, expelling a quantity of coagula, and contracting the uterus to its usual size and firmness; pulse 120, small and weak; complains of giddiness. M.M., perfect rest in horizontal posture; pulverized opium, grs. iii., immediately after rest; panada with brandy. Saw her four hours after; has slept some; no return of 'wasting;' feels comfortable; pulse 100; womb well contracted, no pain on pressure." The case progressed regularly to entire restoration of strength. In another case, the introduction of a piece of ice about the size of the index finger, arrested the hemorrhage, although the placenta did not come away for thirty hours afterwards.

*Nitric Acid.*—Dr. Noble, at a meeting of the "De Witt County Medical Society," stated, in a discussion on the treatment of typhoid fever, that he had saved the life of a patient, who previously had exhibited no alarming symptom, when sinking under passive hemorrhage of the bowels in this fever, by large doses of dilute nitric acid, and found in different cases of this passive intestinal hemorrhage the very best results follow the combination of *turpentine* with nitric acid. Dr. Edmiston united strychnia to the nitric acid in the following proportions:—Strychnia, gr. i.; Nitric acid, ʒi.; Tinct. opii., ʒij.; Aqua, ℥ij.—M. Dose not stated.

---

### ANTHELMINTICS.

*Starving out and Intoxicating a Tænia—Other Remedies.*—A writer, signing himself "L.," who, as he states, is a druggist, gives, in a letter to the editor of the *Am. Journ. of Pharmacy*, an account of the various remedies to which, in his own person, he had recourse for the expulsion of tænia. From his description, he would seem to have suffered at first from ascarides. He was made aware of his having tape-worm by the expulsion, aided by his own efforts at traction, of a portion of this parasite, measuring thirty-two feet in length. In the following year, 1855, he passed "between eighteen and twenty, five feet in length." This was on his return from California. In New Orleans he tried a number of remedies, including koussa, but without any relief. During this time he had a morbid desire for alcoholic liquors, of which he drank very largely. Afterwards, in New York, he substituted bitter tinctures. Finally, after some reflection on his case, he began the following treatment:—When he wanted stimulants, which seems to have been pretty often, he took ten or twelve drinks every day, each consisting of two to three ounces of a mixture of equal parts of the compound tincture of gentian and the tincture of colombo. He took, in addition, only a little soup. At the end of a week he found the worm was getting sick of its diet. It passed off quite freely. We give the rest of the narrative in the words of the writer. "Now, I took a sixteen-ounce syringe, filled it with warm milk and sugar, took the injection, retained it fully two hours; when it passed off, the worm started; I again received over fifteen feet, but before I could pull it all he had fastened. I could feel that it was in the lower intestines, so I did not break it, but held on. I poured out two ounces (fluid) spirits of turpentine, took it with an ounce of castor oil, sat down in a chair for over two hours, (still retaining the worm so as to prevent his going higher;) in a little over two hours the medicine operated, and my friend had got sick of turpentine, and he passed off with the greatest ease, in all a little less than eighteen feet. Fearing there might still be some remains of him, the next day I took one ounce of turpentine followed by oil. This was last April, and I have not felt the least symptoms of the worm since." The letter was written in October. "L." has but a moderate appetite for drink now; in fact, he feels "like a new man."

Some persons may question the safety and necessity of an individual running the risk of becoming an habitual drunkard, in fact of his being temporarily one, in his endeavors to intoxicate the tape-worm in his belly, and at the same time to starve it out. After all, we are inclined to believe that the large dose of turpentine would have been fully operative without the preliminary course.

At a meeting of the St. Louis Medical Society, reported in the *St. Louis Med. and Surg. Journ.*, July, 1857, Dr. Boisliniere presented a specimen of *tœnia lata* or *bothriocephalus* variety, which had been expelled by a dose of koussa after other remedies had failed. Dr. Pollak had treated several patients for tape-worm. In one case the patient was suffering from bilious fever, and was treated with calomel, quinine, and turpentine,—the worm expelled in this case was twenty-nine feet long. In another case he gave turpentine with a drop of croton oil,—the worm expelled was also very long. Dr. McPheters treated with success a case of tape-worm by the administration of a strong infusion of pumpkin seeds: a pint was taken daily until the final expulsion of the tænia. Dr. Pollak suggested as, within his own experience, a preferable plan, to give the pumpkin seeds bruised.

---

### ECBOLICS.

*Ergot of Wheat.*—Dr. McGugin (*Iowa Med. Journ.*, July and August, 1857) relates a case in which, after the expulsion of a fœtus of between three and four months, the placenta was retained, and the os uteri so tightly closed as to preclude resort to any mechanical means for its removal. Ergot of rye was given in drachm doses, but without effect. Dr. McGugin, in running his eye over a field of wheat, saw, on "a number of heads, several parasitic grains similar to, though less than ergot of rye." A large tablespoonful of this was gathered at the doctor's instance, and a decoction was made of it, "which, in less than half an hour after its exhibition, awakened the contractile power of the uterine fibre, and the placenta was thrown off in a decomposed state, after which the woman recovered rapidly." Dr. McGugin adds: the ergot of rye which was used in the first instance had been "pronounced to possess its usual ecbolic properties by others."

*Tartrate of Antimony in Enema.*—Dr. Wm. Read, of Boston, reports (*Boston Med. and Surg. Journ.*, Jan. 1857,) a case of labor of nearly thirty hours' duration, in which the pains were good, but accompanied with no expulsive power; the os uteri was slightly elevated; the membranes had been ruptured spontaneously an hour and a half after the labor had begun. Under these circumstances, about two grains of tartrate of antimony were dissolved in six ounces of luke-warm water, and administered as an enema. "The pains, in a few minutes, entirely changed, becoming exquisite and more frequent; the os dilated fully, a profuse secretion of mucus took place, and the child was born at thirty-five minutes past 1 A.M., with the greatest ease in the delivery."

Shall we compare the operation of the antimony in this case to that of venesection or of opium, by the use of either of which, but under different circumstances, ineffectual expulsive efforts are replaced by strong and effectual ones?

---

### DERMATIATRICS.

*Arsenic and Stillingia in Diseases of the Skin.*—Dr. Wm. M. Cornell, of Boston, read a paper before the Suffolk District Medical Society, July 26th, 1857, which, under the above title, appears in the *Boston Med. and Surg. Journ.*, August, 1857. He speaks of the fluctuations in opinion and practice respecting the use of certain therapeutical agents, and he would lead us to infer that there is reason for a diminution of the favor with which arsenic is still regarded in diseases of the skin of the impetiginous class. He is too general, however, in saying that, "as to cutaneous diseases, arsenic has long been the general constitutional remedy." We would reply that the best dermatologists restrict its use to certain classes of cutaneous diseases, and rest their hopes of its remedial power chiefly in scaly affections, and a few of the more chronic pustular ones.

Dr. Cornell's chief aim, in his paper, is to direct attention to the alterative properties of the *Stillingia Sylvatica*, or Queen's Delight, in a large number of cutaneous and chronic diseases, in which he has given it with decided benefit. He quotes the twenty years' experience of Dr. Simons, and the corroborative testimony of Dr. Frost, both of them eminent practitioners of South Carolina. For a year or two past, he has "given the *stillingia*, prepared from its root as the quinine is from the Peruvian bark. This is given in alterative doses of from one to two or three grains, twice or thrice a day. This is the dose for adults, and proportionately smaller doses for children, according to the age." Dr. Cornell furnishes the following formula for an excellent diet drink.—℞. Stillingia, root recent, ℥iv.; Sarsaparilla, cut fine, lbi.; Spruce shavings, lbiss.; Sassafrass root, ℥vi.; Water, one gallon. Boil in a close vessel a sufficient time to extract the virtues of the articles. Water may be added as it evaporates, and it may be finally reduced to two quarts. To this sugar or treacle is added, and the whole simmered to the consistence of syrup.

"The saturated tincture of the stillingia may be added to the decoction of other ingredients after boiling them, as its virtues consist very much of a volatile principle. The dose for an adult is half an ounce to an ounce."

The efficacy of the stillingia is best manifested in chronic inflammations and other chronic diseases, and more particularly in the tedious sequelæ of syphilis, and in the blood diseases.

*Tannic Acid and Glycerine in Sore Nipples.*—The editor of the *Boston Med. and Surg. Journ.*, October, 1857, is assured by a friend that equal parts, by weight, of glycerine and tannin, is the best application for sore nipples which he has used. It is also an excellent remedy for chaps and excoriations of other parts. The tannin is readily dissolved in the glycerine.

By others a smaller proportion of tannin is introduced into the combination of the two substances.

*Ioduretted Glycerine.*—Dr. Gage, of New Hampshire, as is stated in the *American Druggist's Circular*, recommends this preparation, which has the advantage over both alcoholic and aqueous solutions of not drying. It is made by dissolving one part of iodide of potassium in two parts of glycerine, and with this liquid combining one part of iodine, which becomes completely dissolved. After applying it, the diseased parts thus coated are covered by paper of gutta percha, to prevent evaporation of the iodine.

---

### Topical Stimulants and Escharotics.

*Iodine in Neuralgia.*—Dr. W. G. Thornton relates (*New Orleans Med. News, &c.*, July, 1857) his success in promptly and effectually relieving neuralgia of the left side of the head, by the application of tincture of iodine to the affected part. The effect, says Dr. T., "was almost immediate. I had not finished its application before the pain began to diminish, and soon ceased entirely, and it has not returned since." It may be proper, however, to state that I prescribed immediately—℞. Syrup sarsap. comp., ℥viii.; Potass. iodid., ʒi.—♏. Half an ounce to be given three times a day, and the tincture to be applied next morning in anticipation of an attack.

*Topical Use of Nitrate of Silver for Obstinate Vomiting in Pregnancy.*—Dr. Henry F. Campbell, one of the editors of the *Southern Med. and Surg. Journ.*, in a note prefixed to an article on this disorder, mentions the treatment adopted for its relief, by the now Rev. Dr. C. T. Quintard, formerly Professor of Physiology and Pathology in Memphis Medical College. It consists in cauterizing the fauces freely with a solution of nitrate of silver, consisting of about fifteen grains to the ounce of water. "The experiment was entirely successful." Dr. Campbell asks whether similar means might not act as efficiently in relieving vomiting when it occurs under other circumstances?

A German physician, Dr. Eulenberg, of Coblentz, recommends in strong terms, on account of its efficacy, a very delicate tincture of iodine. He directs ℈i. of the tincture, to ʒiij. of rectified alcohol, of which three drops, in water, are to be taken several times a day. Dr. E. says that other sympathetic irritations and neuroses of the stomach are alleviated by similar treatment.

*Iodide of Zinc in Conjunctivitis.*—A writer in the *Peninsular Journ. of Med.*, March, 1857, calls attention to the use of iodide of zinc as a topical remedy in opacity, resulting from protracted inflammation of the eye. Dr. Pilcher, in his attendance in St. Mary's Hospital, Detroit, "has derived very considerable benefit" from this remedy. "He applies it several times a day in solution, the strength of which is varied according to the sensation produced by it. Ordinarily it is made by dissolving ℈ss. of the iodide in ℥i. of water. Some cases will tolerate a solution twice the strength of this."

---

### ANTIDOTES.

IN the uncertainty as to when a report on *Medical Jurisprudence*, including *Toxicology*, will be made, we give place, at the present time, to a notice of a few alleged antidotes. We say alleged, for it happens, too often, that the first observations, and even experiments, going to show that a particular substance is a certain antidote, are not borne out by succeeding observations and experiments, and hence a justification for extreme skepticism to first announcements of this kind.

*Camphor as an Antidote to Strychnia.*—Dr. Claiborne, of Petersburg, Va., relates the case of a young man about 30 years of age, who took a portion—it is not known how much—of five grains of strychnia, which he had purchased of an apothecary. About forty minutes afterwards, Dr. C. found this person in a state of violent tetanic spasm—touching the bed only with his head and his heels. The convulsions, as they came on, were always tetanic in their form, but often ended epileptic: they were scarcely absent, however, in one or the other form, two minutes consecutively for the first two hours immediately subsequent to Dr. C.'s seeing him, so that the patient was often unable to utter a word for fifteen or twenty minutes at a time. Once or twice, respiration was suspended for so long a time, that Dr. C. feared he had ceased to breathe forever. At these times, a douche of cold water on the face and throat contributed greatly to bring about a successful effort at inspiration. He swallowed with difficulty, even in the intervals of the most complete remission.

The treatment pursued by Dr. Claiborne consisted in the administration of large doses of camphor. He writes: "I gave him, for the first dose, twelve grains of the powdered gum, suspended in a little syrup. In half an hour I repeated the drug, giving him only six grains; and this dose I continued to exhibit every twenty or thirty minutes, taking advantage of the most favorable period of remission, until fifty or sixty grains had been taken. The patient then becoming better, no more was given. No other medicine at all was used during the paroxysm. The day after his attack, I gave him an ounce of the cold-pressed ricini, rubbed up with a little rhubarb, potass., and a few drops of the liq. morph. acetat., and ordered a few dozen leeches to the epigastrium."

Blood in small quantity accompanied the discharge from the bowels for several days, but this did not seem to call for treatment.

Dr. Claiborne thinks it probable that the subject of the above case took between one and two grains of the strychnia.

*Strychnia Poisoning Arrested by Sulphate of Morphia.*—Dr. H. L. Phillips, of Bradysville, Ohio, tells (*Western Lancet*) of his combating with success the poisonous operation of strychnia by sulphate of morphia. A lady, who had been accustomed to use morphia to the amount of two grains at a dose, for a paroxysm of asthma, took, by mistake, in its stead, three grains of strychnia in three doses. Dr. Phillips, on being summoned, lost no time in placing his patient in a warm bath, and in giving her freely the sulphate of morphia. She took of this salt, *five grains* in a period of ten hours. The recovery of the patient was complete.

*Chloroform in Strychnia Poisoning.*—Dr. H. O. Jewett communicates, in the *Boston Med. and Surg. Journ.*, an account of a case of poisoning by strychnia, for which inhalation of chloroform was practised with entire success. The patient, a boy 15 years old, took, by mistake for morphia, a quantity of strychnia, supposed to be about two grains. Thirty or forty minutes passed before aid could be procured. Violent tetanic spasms occurred in quick succession; deglutition was impossible. After the first inhalation of the chloroform, relief was obtained in ten minutes. Partial anæthesia was kept up for about four hours and a half, when it was discontinued. There was no return of the symptoms.

*Ether against Chloroform.*—The asserted antidotal operation of ether against the toxical effects of chloroform, have not been sustained by carefully made experiments on the subject. So far from any beneficially modifying influence being exerted by the ether, it would seem, on the contrary, to have produced a complication more dangerous than the original poisoning.

*Coffee an Antidote to many Poisons.*—Dr. Max. Langenschwarz, in the *Am. Med. Gaz.*, May, 1857, announces, as a fact known to "a very few persons," and, as he believes, "to but a small number of medical men," that "coffee is one of the most important antidotes to many deadly poisons, and to a great many ordinary drugs." Dr. L. directs, for this purpose, "a tincture of raw coffee" prepared as follows:—"Take a quarter of a pound of green coffee (common Domingo the best) and boil it with one quart of water till it is reduced to one pint; then put the whole (berries and liquid) in a quart bottle, add one pint of strong alcohol, and shake it from time to time a little. This tincture gets stronger from day to day, and will, if the bottle is well corked, keep for many years without changing. If to the pint of alcohol (about ten minutes before uniting it with the coffee decoction) you add a little spirits of camphor, say two tablespoonfuls, you will not only triple and double the anti-poisonous quality of the tincture, but this preparation will be an invaluable and certain antidote also to the following poisons:—Garden hemlock, [or dog's] or fool's parsley, (*Æthusa cynapium*,) particularly deadly to full-blooded persons, and producing (by confounding it with common parsley)

almost every year fatal poisonings in all countries; chalk; barytes; poisonous lettuce, (Lactuea virosa;) capsicum, or Spanish pepper; animal coal; cocculus, (Menispermum cocculus;) drosera rotundifolia, or sun dew; euphorbium, spurge, or the so-called wolf's milk; black hellebore; henbane, (Hyoscyamus niger;) hell fig, (Jatropha curcas,) called also the black vomit-nut, one of the most terrible poisons; wild rosemary, (Ledum palustre;) (Moschus,) musk; nitric acid; muriatic acid; phosphoric acid; crows-foot (Ranunculus) of every kind; poisonous snow-rose, (Rhododendron chrysanthum;) garden rue, (Ruta graveolens;) savin, (Sabina,) called also mother tea; ergot, (Secale cornutum;) silica; bittersweet, (Dulcamara;) common sponge, (only the burnt one is employed as a remedy, but I saw very grave accidents to children, having taken pieces of the raw sponge into their mouths;) stavesacre or mice-pepper, (Staphysagria;) tobacco and the horrible nicotine, (the patient can only be saved by our composed antidote of raw coffee, tincture and camphor;) zinc preparations of every kind."

The poisons to which the simple tincture of coffee, prepared as above, is an antidote, as we are assured by Dr. Langenschwarz, are: "laudanum or opium; atropine and belladonna; aconitine and aconite; strychnine and vomit-nut, (*nux vomica;*) chelidonine, and the herb chelidonium majus; caustic lime, potash, and all caustics in general; phosphorus, and all phosphoric preparations; solanine, (principal basis contained in the germs and first shoots of potatoes, and very often self-developed, if potatoes remain moistening in humid cellars;) arum, (Caladium, or Arum seguinum;) brownstone, (Manganum;) veratrin and the white hellebore, (Veratrum album; tansy oil, (or the infusion or tincture of tanacetum vulgare.) The tansy oil has been, and is still recommended by criminal persons, for the purpose of abortion, although it never produced abortion, but frequently kills mother and child together; borax; coloquints; poisoning and suffocation by charcoal vapor, and therefore, of course, also pyrocarbon, (the artificial or chemical development of the same poison; the spotted hemlock, (Conium maculatum.) The conein contained in the seed is one of the most horrible poisons I am acquainted with; iodine; lycopodium; cherry-laurel, (Laurocerasus;) poisonous sumac, (Rhus toxicodendron; valeriana; St. Ignatius's beans; fly mushroom, (Agaricus muscarius;) and all kinds of poisonous mushrooms."

Dr. L. does not favor us with a single experiment in confirmation of his positive assurances of the antidotal power of his tincture of coffee. He proclaims this article itself to be a poison. "If we take a large quantity of boiled coffee without suffering, it is only the habit of taking it from childhood which saves us. People in Turkey take, without danger, quantities of opium sufficient to kill a dozen of us, because they are in the habit of using it from childhood. In Tyrol, the general habit of hunters is, to take a good piece of pure white arsenic upon their tongue, to protect them (so they say) from getting thirsty." Dr. L. does not believe in the identity of cafein and thein, as proclaimed by Liebig; and in this skepticism we join him, on the strength of the marked difference in the effects of tea and coffee on the organism; the former being so grateful to the thirsty and the feverish individual, the latter, on the contrary, as heating and exciting, is regarded with aversion in these conditions of the system. In view of the reactive operations of coffee on certain poisons, which, in proper doses, are given medicinally, this article ought, Dr. L. tells us, to be withheld when we desire to place the patient under the influence of the latter substances. "If, for example, a patient swallows, at seven o'clock, a spoonful of iodine preparation, and then takes, ten minutes after, only one spoonful of good coffee, he will at half-past seven have no more iodine in force in his system than he has whalebone force in his hairs. But," adds the author, in a fling at dishonest grocers, "certainly the matter I speak of must be coffee, and not that innocent and precious genuine coffee article of certain New York merchants, consisting one-half in roasted carrots, another half in succory, and (excuse that new arithmetic) a third half in 'smart merchants' tricks.'"

As relates to dose and mode of administration, Dr. Langenschwarz gives the following directions:—"The compound saving-tincture (of green coffee and camphor) is, in the respective cases of poisoning, to be administered naturally and by clyster; the internal dose, about ten to twelve drops in a teaspoonful of water every five minutes, and every fifteen minutes when the patient begins to recover. Larger, and even very large doses may be given if the danger of life is imminent."

*Bibron's Antidote to the Poison of the Rattlesnake.*—Dr. Hammond, United States Army, relates (*Am. Journ. Med. Sci.*, Jan. 1858,) the results of his observations and experiments on July 1, 1857, to show the value of this antidote. The first case was that of a hospital steward, bitten by a rattlesnake on the inner finger of the right hand. About four minutes after the bite, Dr. H. administered Bibron's antidote, after which the symptoms almost immediately disappeared. The pain and swelling, with throbbing, of the part, having returned after a period of forty minutes, the doctor repeated the medicine, and in less than five minutes the finger had regained its natural appearance, and all pain and pulsation had vanished. Two experiments on animals, the one on a wolf, the other on a dog, both of them bitten by a rattlesnake, showed the great activity of the antidote in arresting, at once, the alarming symptoms and restoring the animal to health.

Professor Bibron's antidote is, according to Prince Paul of Wurtemberg, who took a lively interest in the matter, prepared as follows:—Potassii. iodidi, grs. iv.; hydrargy. chlorid. corros, grs. ii.; bromini, ʒv.—♏. Ten drops* of this mixture, diluted, with a tablespoonful of wine or brandy, constitute a dose, to be repeated if necessary. It must be kept in a glass-stoppered vial, well secured.

Dr. Coolidge, United States Army, communicates to Dr. Hammond an account of the case of a girl, aged 15 years, who was bitten in July, 1857, at Fort Riley, by a rattlesnake, on the dorsal aspect of the first phalanx of the ring finger of the right hand. In a few minutes the finger became swollen and blueish, and in about ten minutes after receiving the wound, when Dr. Coolidge first saw her, the forearm had begun to swell, and pain extended to the elbow. The patient was depressed and somewhat nauseated. An older sister had sucked the wound at the very first instant of its infliction. Dr. C. made a free incision down to the bone, and as soon as the articles could be prepared, he gave ten drops of the bromine mixture, diluted, and injected into the wound an iodu-

---

* As no solvent is mentioned in this formula, we are unable to attach any meaning to the dose of ten drops, or to tell its strength.

retted solution, recommended by Dr. David Brainard, consisting of iodine, ten grains; iodide of potassium, thirty grains; and distilled water, one ounce. This caused severe smarting pain. The patient expressed herself relieved after the first dose of the bromine; a second one was given in twenty minutes; nothing more was done; the girl recovered,

*Lard an alleged Antidote to Strychnia.*—As a practical commentary on our introductory remarks on antidotes, we may repeat here the results of Dr. Hammond's experiments on animals, with a view of testing the antidotal virtues of lard, as set forth by Dr. Pindels. Dr. H.'s conclusion is, that if it have any effect, it is to give increased activity to the toxical operation of strychnia. He gave to two dogs each two grains of strychnia, and to one of them, in addition, a pint and a half of lard. The dog which took the lard died; the other, a miserable cur, escaped unharmed.

Shall we introduce, under the head of Antidotes, Vaccine Matter against Smallpox—not as a preventive, but, pending this latter disease in its declared state, as a cure? The fact, if fact it be, is announced in the *Am. Journ. of Med. Sci.*, Oct. 1857, in the following terms:—

"*Vaccine given Inwardly for the Cure of Smallpox.*—By R. Landell, M.D." This gentleman first tried this new treatment in epidemic smallpox in 1842, and between this date and 1854 he has had every reason to be satisfied with its success. His directions are, to "dissolve the vaccine that is contained on a pair of plates in a capillary tube, which is about four or six drops of vaccine lymph, in four or six ounces of cold water, and give to the patient a tablespoonful every two or three hours."

"The favorable result of this exhibition is, that it mitigates the symptoms, modifies the species, and cures the smallpox." More than this no reasonable man can expect. Dr. L. tells us, in addition, that, by this treatment, he has "seen disappear the fever, delirium, hoarseness, diarrhœa, pneumonia, cerebral congestion, and the secondary fever."

The cure is not, however, left entirely to the vaccine matter, as we learn from the following:—"It may be mentioned here that the use of emollient clysters or castor oil internally, to keep the bowels loose, and in children, calomel, is very necessary; as also gargles of nitrate of silver and chloruret of lime. And, after the fifth day, give baths of warm water, with a little chloruret of lime, or chloruret of soda, or sponge the body."

Dr. L. has, also, "given vaccine inwardly as a therapeutic remedy in hooping-cough."

Hitherto, we have been taught to believe in the general innocuousness of animal poisons, when introduced into the stomach. If Dr. Landell's treatment be confirmed by the experience of others, we must, for the future, abandon this creed as untenable. The gentleman writes from *Porte Alegre*, Brazil.

There are yet other papers, on subjects some of them actually belonging, others germain to our branch, but we can only barely allude to them now, as we have already far exceeded the limits originally assigned to us. Among the therapeutical articles we may mention *Medical Inhalation*, some useful observations on which are made in the *Bost. Med. and Surg. Journ.*, Oct. 1857, by Dr. E. J. Coxe, Visiting Physician of the Charity Hospital, New Orleans. He had discussed the subject, in greater detail, in a former number of the *Boston Journal.* Attention may also be directed to an ingenious essay on the "*Use of Water in the Treatment of Fever*," by Dr. Isaac Casselberry, of Indiana, in the *Am. Journ. of Med. Sci.*, July, 1857. Of a similar tenor, but deficient also in clinical application, is "*An Inaugural Dissertation on Cold Water as an Auxiliary in the Treatment of Disease*," submitted for the degree of Doctor of Medicine in the University of Nashville, and published in the *Nash. Journ. of Med. and Surg.*, March, 1857. An instructive article, illustrated by numerous wood-cuts, "*On the Localization of Baths, and on the Application of Cold and Heat on the Different Parts of the Body*," by Charles Mayor, M.D., will be found in the March number of the *N. Y. Journ. of Med.*, 1857.

It is lamentable to think of the neglect, by the great body of the profession, of the curative powers of water, externally applied, in a long list of diseases, including more particularly those of the febrile class. As an agent of hygiene, it is, when not neglected, too often misapplied by the people at large; nor are they much benefited by the crude advice, growing out of ignorance of the subject, by so many of their medical advisers.

Remarks to the same purport might be made in reference to the waters of Mineral Springs, the use of which, in a great majority of cases, is purely empirical.

Dr. Wright, Professor of Chemistry in the Kentucky School of Medicine, has a paper in the *Western Lancet*, May, 1857, "*On the Chemical Composition and Medical Properties of the Waters of Grayson Springs, Ky.*" In 10,000 parts, by weight of the water, he found, on an average, 1270 of different saline substances.

We meet with some papers in the Journals through which we have looked, on the *Materia Alimentaria;* such, for example, as the "Report on *Condensed Milk*, by the Section on Materia Medica and Botany" of the N. York Academy of Medicine, published in the *Am. Med. Gaz.*, Dec. 1857, a synopsis of which appears in the *N. Y. Journ. of Med.*, Jan. 1858. We indulge the hope that, on a future occasion, a reasonable space will be given to a consideration of the whole subject of Materia Alimentaria.

Dr. Huff, of Lexington, Ky., has a short and instructive paper "*On the Use and Abuse of Chemical Baths*," which appears in the *N. Y. Medical Times.* Dr. H. explains the marvellous accounts of mercury being eliminated by galvanic process from the bodies of those who had previously taken this metal to any extent. He shows, that what has been mistaken for mercury is tin eliminated from the metallic bath-tub, and deposited on a plate of copper within the said tub.

We notice with pleasure an article, which is a continuation of a preceding one, "*On Hygiene*," by A. B. T., in the *Medical Independent*, for April, 1857.

As coming within the scope of our present theme, mention may also be made of the labors of Dr. J. P. Batchelder, of the City of New York, on enlisting gymnastics for the cure of paralytics, of which notice is taken in the *Medical and Surgical Reporter*, (1857.)

